# Structure and Preliminary Reliability of the Diet Quality Questionnaire (DQQ)-Based Form Adapted for Use in the Polish Population—Results from Initial Validation Stage

**DOI:** 10.3390/nu18071044

**Published:** 2026-03-25

**Authors:** Paweł Rzymski, Agnieszka Zawiejska, Katarzyna Tomczyk, Alicja Rzymska, Małgorzata Kampioni, Agnieszka Lipiak, Małgorzata Kędzia, Ewelina Chawłowska, Beata Pięta

**Affiliations:** 1 Department of Mother’s and Child’s Health, Gynecologic and Obstetrical University Hospital, Poznan University of Medical Sciences, Polna St 33, 60-535 Poznan, Poland; 2 Department of Preventive Medicine, Poznan University of Medical Sciences, 60-781 Poznan, Polandewierz@ump.edu.pl (E.C.); 3 Department of Reproduction and Gynecology, Gynecologic and Obstetrical University Hospital, Poznan University of Medical Sciences, 61-701 Poznan, Poland; 4 Students’ Scientific Society, Poznan University of Medical Sciences, 60-781 Poznan, Poland; 5 Department of Practical Midwifery Education, Poznan University of Medical Sciences, 60-781 Poznan, Poland

**Keywords:** Diet Quality Questionnaire (DQQ), cultural adaptation, test–retest reliability, Cohen’s kappa, dietary diversity (DDS), Global Dietary Recommendations (GDR) score, population surveillance, polish adaptation, linguistic validation

## Abstract

**Background/Objectives:** The Diet Quality Questionnaire (DQQ) is a brief, food group–based instrument designed for globally comparable population surveillance of diet quality. We culturally adapted the DQQ for Poland and evaluated its internal structure and reliability in an adult cohort. **Methods:** Following forward–backward translation and expert review, the Polish DQQ was administered online to adult females. Internal structure was explored and test–retest reliability was assessed for total DQQ scores. Diet quality indicators (Dietary Diversity Score [DDS], NCD-protect, NCD-risk, and Global Dietary Recommendations score [GDR]) were summarized descriptively. **Results:** The average age in the cohort was 29.4 ± 13.6 years. A total of 296 respondents completed the survey; 100 completed the retest. Item-level test–retest reliability was good to excellent (Cohen’s kappa 0.72–1.00). Agreement for total scores was high with minimal bias (Bland–Altman bias 0.2, >95% of observations within limits of agreement) and there was no heteroscedasticity; Passing–Bablok regression indicated equivalence between the test and retest. Median (IQR) diet quality indicators were: DDS 6.0 (5.0; 7.0), NCD-protect 2.5 (1.5; 4.0), NCD-risk 2.5 (1.0; 4.0), and GDR 9.0 (7.5; 10.5). Eighty percent met DDS ≥ 5, while one-third consumed all five recommended food groups. **Conclusions:** DQQ-PL demonstrates high item-level stability and strong agreement for total scores, with structural findings aligning with its design as a non-latent, food group checklist for population monitoring. The Polish adaptation is feasible and reliable in the studied population (young adult women), supporting its potential use for rapid dietary surveillance pending broader validation.

## 1. Introduction

Diet quality is a major modifiable determinant of population health and one of the leading risk factors for non-communicable diseases (NCDs), including cardiovascular diseases, type 2 diabetes, and certain cancers. The burden of diet-related chronic diseases remains substantial, highlighting the need for standardized tools enabling reliable population-level dietary monitoring. Traditional dietary assessment methods, such as quantitative 24-h dietary recalls (24HRs) and food frequency questionnaires (FFQs), provide detailed intake data but are resource-intensive due to the time-consuming nature, methodological complexity and procedural requirements of traditional assessment methods [1,2,3]. These methodological and logistical constraints limit their feasibility for large-scale, harmonized global surveillance.

To address this gap, the Diet Quality Questionnaire (DQQ) was developed as a standardized, food group-based instrument designed for global use. The DQQ applies a 24 h reference period but does not collect quantitative intake or portion size data. Instead, it uses 29 dichotomous (yes/no) questions covering sentinel foods representing key food groups, enabling calculation of diet quality indicators related to protective and risk components of NCDs. Cognitive testing across multiple languages demonstrated that the sentinel food approach improves comprehension and reduces misclassification compared with open-ended dietary recall methods [1,2,3]. The Global Diet Quality Questionnaire (DQQ) is a tool developed by researchers within the Global Diet Quality Project to collect information on dietary intake across various food groups, enabling assessment of nutritional quality among the study populations. Its straightforward structure enables widespread use online, among the general population, and worldwide.

Unlike composite indices such as the Healthy Eating Index (HEI) or Mediterranean diet adherence screener like MEDAS (Mediterranean Diet Adherence Screener) or MedQ-Sus (Mediterranean Diet and Nutritional Sustainability Questionnaire, short Mediterranean diet questionnaire), the DQQ does not require portion estimation or nutrient calculation, making it particularly suitable for population-level monitoring and cross-country comparisons [4,5,6]. The questionnaire has been adapted as a Global Public Good and implemented in more than 140 countries following a standardized cultural adaptation protocol [3].

In Poland, dietary assessment tools such as KomPAN (Dietary Habits and Nutrition Beliefs Questionnaire), WOBASZ II (National Multicentre Health Survey) and various FFQ-based instruments are available [7,8]. However, no harmonized, internationally comparable, food group-based short instrument has been culturally adapted and psychometrically validated for the Polish population. Additionally, nationally developed questionnaires are not recognizable worldwide [1,3].

In light of the increasing population burden of diet-related diseases, there is a need for a standardized, comparable instrument to assess diet quality across research and surveillance settings—the Diet Quality Questionnaire (DQQ) version suitable for the Polish context has not been developed or validated to date. Therefore, the aim of the present study was to conduct a full cultural adaptation of the DQQ using a forward–backward translation protocol and assess its psychometric performance, focusing on internal consistency and test–retest reliability, in an adult cohort.

## 2. Materials and Methods

The principal investigator approached the Global Diet Quality Project team and obtained approval to use the tool in the Polish population. The study protocol was reviewed and approved by the Bioethics Board at the Poznan University of Medical Sciences (Approval No: KB 136/26).

The original Diet Quality Questionnaire (DQQ) was developed by Herforth et al. from well-established, globally recommended indicators and scales of nutrition quality, including the Minimum Dietary Diversity for Women (MDD-W) and the All-5 questionnaire [1]. It consists of 32 binary (yes/no) items that ask whether a person completing the survey consumed specific foods the previous day. The “yes” responses are summarized and these raw data allow the computation of additional indices of diet quality as the sums of specific items: Dietary Diversity Score (DSS; maximum value: 10), consumption of the foods associated with the lower risk of non-communicable disorders (NCD-protect score; maximum value: 9.0), consumption of the foods associated with an increased risk for non-communicable disorders (NCD-risk score; maximum value: 9.0) and consumption of appropriately varied foods (Global Dietary Recommendation Score; GDRS computed from the following formula: NCD-Protect − NCD-Risk + 9) [1]. Following recommended phased approaches to instrument validation, this study focused on evaluating the reliability and structural characteristics of the Polish DQQ. Criterion validation against reference methods (e.g., multiple-pass 24 h recall or observed weighed food record) will be conducted in the next phase using a probability-based adult sample.

To develop a Polish version of DQQ (DQQ-PL), the English version was translated into Polish and back-translated by a bilingual individual with a professional background in health sciences, who was blinded to the original DQQ. The translation process was conducted according to the WHO guidelines [9]. Finally, all discrepancies were discussed by the authors and expert team, comprising researchers with sufficient English proficiency and experience in nutrition research to ensure that clarity and cultural context were appropriately addressed. Having fully agreed on the final version of the DQQ, the online survey was prepared for subsequent use. Details are provided in Appendix A.

Next, the Polish translation of the questionnaire was administered as an online survey and distributed via social media or SMS, using convenience sampling. To obtain data for calculating test–retest reliability, all participants who completed the survey once were asked to complete it again 6 h later, to reflect the previous-day dietary structure. Overall, 296 participants completed the survey once, and 100 participants completed both the test and the retest survey. The data collection was performed between 1 December 2025 and 15 January 2026.

Inclusion criteria for the validation study include females aged 18 years and older. Participants were recruited via Polish social media platforms through a general, non-targeted advertisement. The announcement invited individuals to voluntarily participate and provided instructions for completing the DQQ-PL questionnaire. The survey was administered online, accessible on both mobile and desktop devices, and the data-collection interface required respondents to answer every item, thereby preventing missing responses. The recruitment announcement was disseminated nationwide through open social-media channels and SMS, making it accessible to residents from rural areas, small towns, and large urban centers. Although this approach offered the potential to reach a geographically diverse audience, the final sample still reflects a convenience-based composition. Participants were additionally asked to provide a unique self-generated code to enable linkage between baseline and follow-up assessments for the test–retest subsample. This procedure ensured that questionnaires were both complete and correctly matched across repeated administrations.

### Statistical Analysis

To assess the structure of the DQQ in the population studied, we first examined the tetrachoric correlation matrix, which is appropriate for binary covariates. Visual analysis of the matrix plot, completed with the KMO test, which yielded an overall MSA of 0.08, confirmed no factorial structure of the dataset. Therefore, we opted for a data-driven approach and examined the DQQ structure in our population using non-hierarchical cluster analysis (k-means clustering).

In the subset of the study group who completed the DQQ twice, an unweighted Kappa was computed to assess test–retest reliability, and a Bland–Altman plot with Passing–Bablok regression was used to examine agreement between test and retest raw DQQ scores [10,11,12]. The statistical analysis was performed using R Program (version 4.5.2, Software, PBC, Boston, MA, USA).

## 3. Results

### 3.1. Descriptive Characteristics

The average age in the cohort was 29.4 ± 13.6 years. Participants who provided data for a retest analysis were significantly younger than those who completed the DQQ survey only once (mean age difference: 8.6 years; 95% CI: 5.5–11.7 years). Compared with participants who completed the DQQ only once, those who completed the DQQ twice responded “yes” to questions 9, 16, and 23 significantly less frequently during the first round of the survey.

Table 1 summarizes the distribution of the responses to the DQQ items in the cohort.

Table 2 summarizes the descriptive statistics for diet quality indices in the cohort. Participants who took the retest did not differ from those who did not on DSS, NCD-protect, NCD-risk, and GDRS.

Overall, the characteristics indicate average-to-good dietary diversity, with the majority of participants meeting the criteria of adequate micronutrient intake (DSS above five in over 80% of the cohort).

However, only one-third of the cohort consumed a recommended variety of foods, measured as having foods from all five critical food groups every day. While almost all participants reported having animal-source foods in the previous days, and almost 90% of the cohort reported having at least one vegetable or at least one starchy staple food the previous day, less than half of the cohort confirmed having at least one portion of pulse, nut, or seed when surveyed.

### 3.2. Structure of the DQQ in the Cohort

**Figure 1:**
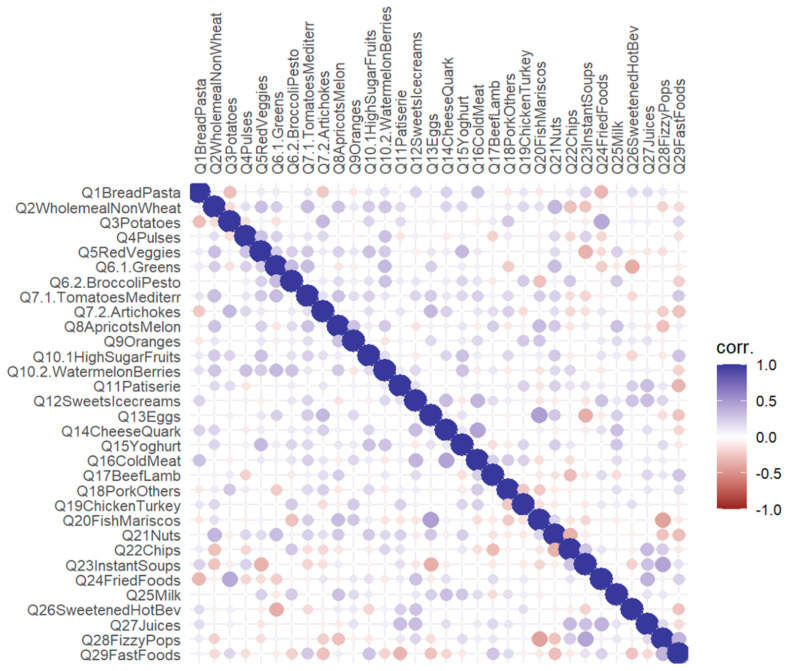
A matrix for tetrachoric correlations in the cohort.

The matrix plot of tetrachoric correlations, as shown in Figure 1, indicates no clear factor structure in the survey, and between-item correlations are predominantly weak or lacking.

**Figure 2:**
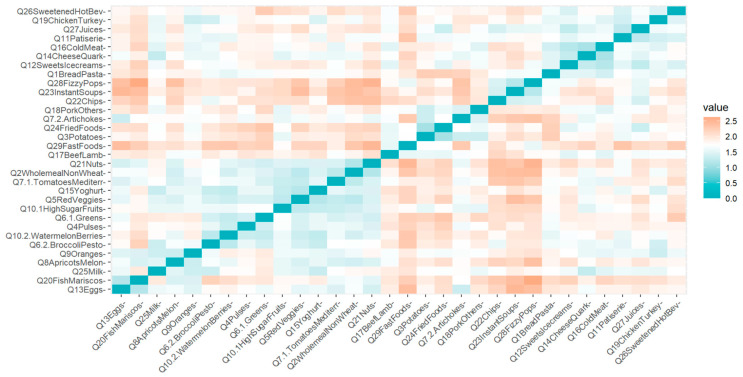
A distance matrix for the cohort.

To explore the survey structure in our population, we performed k-means clustering. Figure 2 presents a distance matrix for our cohort. Overall, the visual analysis of the matrix indicates a lack of distinct blocks of covariates, and the visual inspection of the distance matrix did not reveal any meaningful or well-defined clustering patterns, which is consistent with the expected non-latent structure of the DQQ.

**Figure 3:**
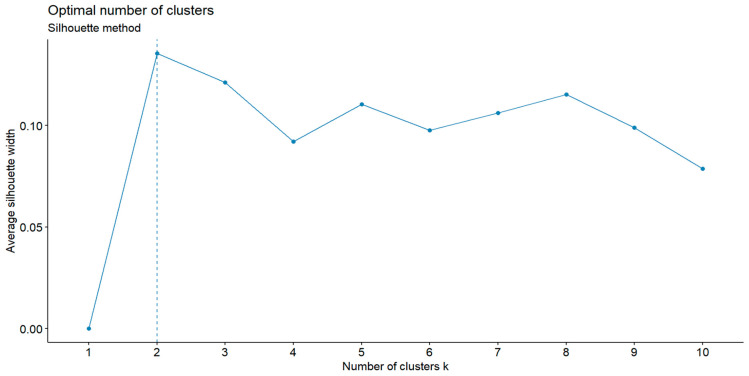
Identification of the optimal number of clusters with the silhouette method.

Diagnostic tests identified between 1 and 10 clusters as an appropriate structure of the dataset, with 2 clusters identified by a majority (9 out of 22, 40.9%) of the methods. The results of the analysis using the silhouette method are shown in Figure 3. However, the metrics used to evaluate clustering quality returned low values, indicating poorly defined clusters (silhouette index = 0.14; Calinski–Harabasz index = 6.58).

**Figure 4:**
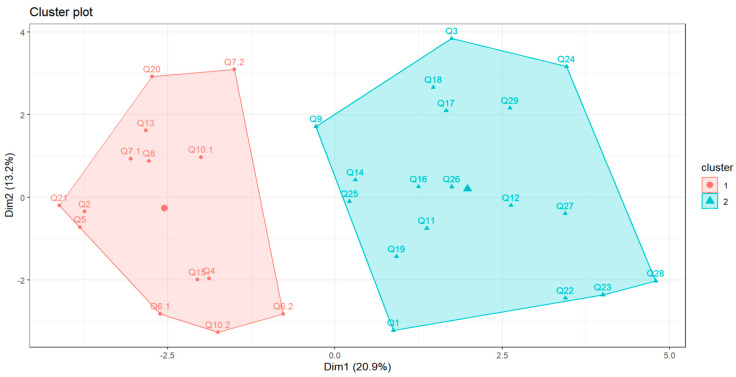
A two-cluster structure of the DQQ in the cohort.

Figure 4 shows the two-cluster solution returned by the algorithm; however, given the very low silhouette index, these clusters should not be interpreted as substantively meaningful.

### 3.3. Test–Retest Reliability of the DQQ

Table 3 presents Cohen’s kappa coefficients for the items. Overall, the analysis indicates good to excellent agreement, with Cohen’s kappa coefficients for individual items ranging from 0.72 for items DQQ.1 and DQQ.7.2 to 1.0 for items DQQ.20 and DQQ.23.

### 3.4. Agreement Between a Raw Test and a Retest Scoring

**Figure 5:**
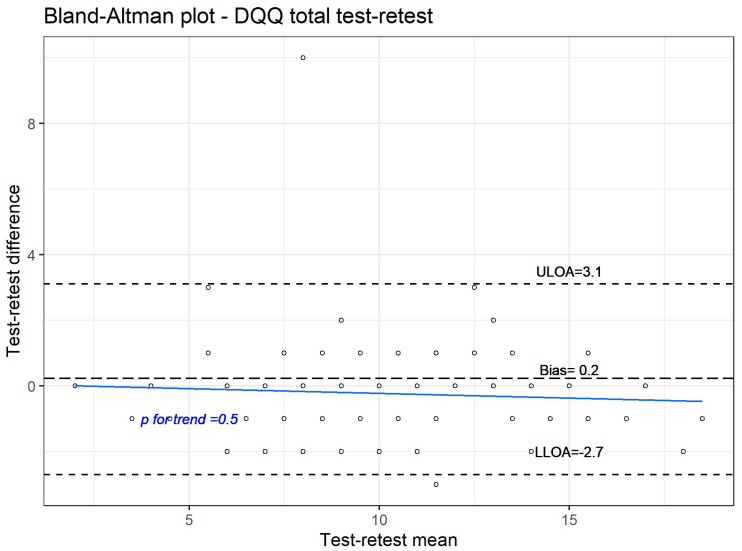
Agreement between a test and retest total DQQ scoring.

To examine the agreement between the total scores obtained in a test and a retest, we plotted the difference between the total DQQ test and retest scores against the mean of these two scores—this plot, also known as the Bland–Altman plot, is shown in Figure 5 [10,11,12]. The analysis confirmed a very good agreement between the test and retest DQQ scoring. We observed a very small bias (0.2), with more than 95% of measurements falling within the limits of agreement, and the absence of a significant trend indicated that the data exhibited no heteroscedasticity.

**Figure 6:**
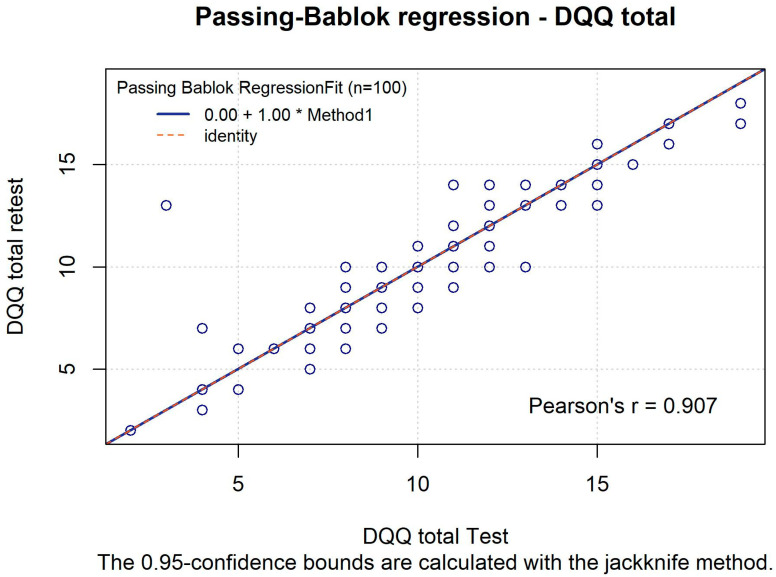
Passing–Bablok regression model for a test vs. retest total DQQ scoring.

We confirmed these observations by fitting a Passing–Bablok regression model, which indicated a perfect comparability of the test and retest measurement (Figure 6).

## 4. Discussion

Dietary assessment remains one of the most methodologically challenging domains in nutritional epidemiology. As highlighted in the review by Cade et al. [13], food frequency questionnaires (FFQs), despite their widespread use, are associated with substantial respondent burden, recall bias, misclassification, and measurement error. Moreover, FFQs require extensive food composition databases, portion size estimation and complex nutrient calculations, which increase both financial and logistical costs and may limit their feasibility in large-scale monitoring. Short adherence screeners such as MEDAS are focused exclusively on Mediterranean diet patterns and do not capture the broader multidimensional aspects of overall diet quality. While FFQs remain valuable for etiological research, their routine use in national surveillance is constrained by these methodological demands [4,5,6,13].

In contrast, the DQQ was specifically designed to address these limitations. Unlike traditional quantitative dietary recalls or FFQs, the DQQ applies a simplified 24 h reference period and dichotomous (yes/no) food group reporting without requiring portion size estimation or nutrient content. The sentinel food approach further reduces cognitive burden and improves cross-cultural interpretability, as demonstrated in cognitive validation studies conducted across multiple languages [2]. This structure makes the DQQ particularly suitable for rapid deployment in diverse sociocultural settings and large epidemiological surveys. From a nutritional epidemiology perspective, the DQQ offers a unique combination of brevity, low implementation cost, and international harmonization that is not achievable with conventional FFQs or disease-specific adherence indices [1,2,3,14,15]. In contrast to traditional FFQs, which typically require 20–60 min and generate detailed quantitative intake data, the DQQ focuses on the presence of key food groups and achieves cross-country comparability across more than 140 language versions through the use of sentinel foods [3].

Beyond its rapid uptake, the global use of the Diet Quality Questionnaire (DQQ) demonstrates how a modern dietary assessment instrument can achieve both standardization and contextual flexibility—two features historically difficult to combine in nutrition epidemiology [3]. Recent studies illustrate this dual advantage: DQQ has been validated or applied in African settings, including Rwanda [14], Ghana [16,17], Ethiopia, Vietnam and the Solomon Islands [18]; across multiple South and Southeast Asian countries such as Bangladesh, Pakistan, and India and extensively throughout China [15,19,20,21], where it has been used to study depression, anxiety, stress, cognitive decline, dietary supplement use, school-based mental health, and pediatric obesity. The tool has also been tested in Latin America, for example, in Chile, where sentinel foods and Global Dietary Recommendation indicators were validated in pregnant women and children [22]. The detailed global application in most recent studies is presented in Table 4. This breadth of applications highlights the DQQ’s universal structure—built on 29 standardized food groups and locally adapted sentinel foods—which allows researchers to generate comparable diet quality indicators across countries while still capturing culturally specific dietary patterns. Its short administration time, minimal respondent burden, and compatibility with both in-person and digital data collection make it uniquely suitable for large population surveys, vulnerable groups, school-based interventions, social protection evaluations, and behavioral or mental health studies. As the literature shows, DQQ has evolved rapidly from a methodological innovation to a widely validated global standard, facilitating harmonized nutrition monitoring in an unprecedented range of scientific and programmatic domains [3,14,15,16,17,19,20,22,23,24,25].

Nevertheless, several limitations should be acknowledged. Our study employed online administration, which may introduce mode-specific constraints such as coverage and self-selection. However, the DQQ was purpose-built for low-burden population surveillance and has been successfully deployed in national, probability-based surveys, where self-administered and remote formats are standard and designed to maximize reach and comparability across settings. Recent methodological publications support self-administration as a valid modality for diet quality monitoring and provide cognitive validation of DQQ’s food group questions across languages, suggesting that reliable data can be obtained without face-to-face interviews. As a qualitative food group-based instrument, the DQQ does not provide quantitative nutrient intake estimates and therefore cannot replace detailed dietary assessment in etiological or clinical research. Furthermore, although the 24 h reference period minimizes long-term recall bias, it may not fully capture habitual dietary patterns at the individual level. However, for population-level surveillance, repeated cross-sectional administration is considered methodologically appropriate and consistent with its intended use. Additionally, the original dietary diversity and diet quality metrics from which the DQQ was derived were initially validated in women of reproductive age. Although the DQQ has since been used internationally across sexes and age groups, this historical origin should be acknowledged as part of the tool’s developmental context. The primary objective of this study was linguistic–cultural adaptation and short-interval reliability testing of the Polish DQQ, prioritizing fidelity to the original instrument and clarity/comprehension in the target language. Accordingly, we used a convenience sample to evaluate measurement properties (item comprehension, stability, agreement) rather than to estimate population-level diet quality. As such, our findings should not be interpreted as nationally representative, nor as directly generalizable to men or older adults. This study constitutes the first step in a staged validation program; subsequent work will employ probability-based sampling across sexes and age groups (and, where feasible, mode comparisons and external references such as 24 h recalls) to support population surveillance and subgroup analyses. The current evidence addresses how well the Polish DQQ measures, not how the Polish population eats; the latter will be the focus of future, representative studies. Additionally, any visual clustering tendencies observed in the distance matrix should be interpreted as an early, purely descriptive observation without analytical significance, given the weak clustering metrics and the non-latent design of the DQQ.

In our validation study, we observed a statistically significant age difference between the main validation sample (N = 296) and the smaller test–retest subsample (N = 100). Such discrepancies are common in questionnaire validation because re-contact and repeat participation typically attract a more “responsive” subset—often slightly younger and more digitally engaged—without implying a systematic selection bias. Methodological work on repeatability shows that test–retest subsamples are frequently smaller and demographically skewed due to voluntary re-enrolment, yet this does not threaten reliability estimation provided the retest group remains within the instrument’s intended user population and key construct-relevant characteristics are preserved. Our instrument (DQQ) captures the presence/absence of food group consumption for the prior 24 h. Because the referent day changes with longer gaps, extending the retest to 1–14 days would plausibly conflate true intake variation with measurement error, thereby biasing reliability downward. This is a well-recognized dilemma in test–retest design: shorter intervals reduce the risk of genuine change, whereas longer intervals reduce memory/carryover but increase construct drift; the appropriate choice depends on how stable the construct should be between administrations. In tools explicitly framed as “previous-day” recalls, same-day or very short-interval retest has precedent and has yielded acceptable item-level agreement (kappa) when the goal is to isolate instrument stability rather than habitual-diet estimation [26,27]. Comparable patterns have been reported in validations of diet quality instruments: for FFQ-derived indices (AHEI, DASH, aMED), demographic differences between those completing both waves versus the full baseline did not compromise reproducibility or validity; similarly, in the short Mediterranean diet questionnaire (MedQ-Sus), the retest subset differed demographically (including a higher proportion of younger respondents) without undermining reliability conclusions. Test–retest assesses the stability of the tool, not the comparison of two groups. Critically, because the DQQ captures the presence of food groups over the prior 24 h rather than age-sensitive latent traits, modest age differences between the validation and retest samples are methodologically acceptable for assessing stability and should be framed as an expected by-product of voluntary repeat participation in longitudinal instrument testing [6,28,29,30]. Although the present study did not include a comparison with a reference method, extensive international evidence demonstrates high criterion validity of the Diet Quality Questionnaire. Studies conducted in Ethiopia, Vietnam and the Solomon Islands show very small differences between DQQ and 24 h dietary recalls (0.6–2.5 percentage points in food group prevalence) and high percent agreement (approximately 89–96%), with comparable FGDS, NCD-Protect, NCD-Risk and GDR scores across methods. Furthermore, validation in Rwanda using same-day DQQ, 24hR and observed weighed food records reported high agreement and minimal, non-significant differences relative to Observed Weighed Food Record (OWFR); notably, in this study, the 24hR underestimated MDD-W relative to OWFR more strongly than the DQQ. These findings indicate that DQQ performs robustly at the population level compared with conventionally used reference methods. At the same time, we acknowledge that criterion validity must be established specifically for the Polish context. Therefore, we frame our current conclusions as the first step and have planned a follow-up validation phase incorporating repeated 24 h measurements and, where feasible, OWFR or context-driven applications [14,18].

## 5. Conclusions

This study provides the first cultural adaptation and psychometric evaluation of the Polish version of the Diet Quality Questionnaire (DQQ). Our findings suggest that the Polish DQQ demonstrates satisfactory reliability and structural coherence, supporting its potential utility as a rapid dietary surveillance tool in population-based research. Overall, our findings support the feasibility and reliability of the DQQ as a standardized, rapid dietary assessment tool. Its implementation may enhance the monitoring of diet-related risk factors and contribute to internationally comparable nutrition surveillance data.

## Figures and Tables

**Table 1:** Frequency of “yes” responses to the DQQ items in the cohort (the test DQQ survey).

DQQ Item	*N* (%) of “Yes” Responses
Q1 Pasta, ravioli or tortellini, rice, bread, focaccia, pizza, piadina, or crackers?	203 (68.6)
Q2 Barley, spelt, whole grain pasta, polenta, muesli, or popcorn?	72 (24.3)
Q3 Potatoes or gnocchi?	104 (35.3)
Q4 Beans, chickpeas, fava beans, lentils, peas, tofu, or soymilk?	59 (19.9)
Q5 Carrots, pumpkin, or red bell pepper?	151 (51.0)
Q6.1 Spinach, kale, arugula, escarole, chard, watercress, or chicory?	80 (27.0)
Q6.2 Basil pesto, broccoli rabe, or broccoli?	45 (15.2)
Q7.1 Tomatoes, eggplant, zucchini, mushrooms, radicchio, fennel, or lettuce?	197 (66.6)
Q7.2 Artichoke, asparagus, cabbage, cauliflower, beets, green pepper, or green beans?	70 (23.6)
Q8 Apricot, dried apricot, loquat, canteloupe, or persimmon?	18 (6.1)
Q9 Orange, mandarin, clementine, or grapefruit?	122 (41.2)
Q10.1 Dried or fresh figs, dried or fresh plums, grapes, peaches, apple, pear, or banana?	134 (45.3)
Q10.2 Watermelon, honeydew melon, kiwi, pomegranate, strawberries, cherries, or berries?	44 (14.9)
Q11 Cakes, biscuits, merendine, cannoli, pastries, donuts, or tart?	121(40.9)
Q12 Candy, chocolates, gelato, semifreddo, tiramisu, panna cotta, custard, or chocolate pudding?	114 (38.5)
Q13 Eggs?	146 (49.3)
Q14 Cheese or fresh cheese?	195 (65.9)
Q15 Yogurt?	134 (45.3)
Q16 Cured meats such as prosciutto, mortadella, salted beef, salami, pork belly, or dried sausage?	158 (53.4)
Q17 Beef, veal, lamb, sheep, goat, or meatballs?	36 (12.2)
Q18 Pork, rabbit, or horse?	50 (16.9)
Q19 Chicken or turkey?	151 (51.0)
Q20 Fish, canned fish, anchovies, sardines, octopus, or other, seafood?	47 (15.9)
Q21 Walnuts, hazelnuts, pistachios, almonds, cashews, or peanuts?	109 (36.8)
Q22 Chips such as San Carlo, Fonzies, or Pringles?	28 (9.5)
Q23 Instant Ramen?	7 (2.4)
Q24 French fries, fried fish, fried vegetables, fried veal cutlet, fried meatballs, fried meat pastry, or chicken nuggets?	59 (19.9)
Q25 Milk?	168 (56.8)
Q26 Sweetened coffee, tea with sugar, hot chocolate, or chocolate drinks?	98 (33.1)
Q27 Juice, fruit flavored drinks, or fruit syrup?	74 (25.0)
Q28 Soft drinks such as Fanta, Coca-Cola, or Sprite, energy drinks, or sports drinks?	69 (23.3)
Q29 McDonald’s, Burger King, KFC, or other place that serve burgers and french fries?	9 (3.0)

**Table 2:** Indicators of diet quality in the cohort; data shown as median (25th, 75th percentile) or as number (%).

Indicator		
Dietary Diversity Score (DSS)		6.0 (5.0; 7.0)
Participants with adequate micronutrient intake (DSS >= 5)		238 (80.4)
NCD-protect score		2.5 (1.5; 4.0)
NCD-risk score		2.5 (1.0; 4.0)
Global Dietary Recommendations (GDR) Score		9.0 (7.5; 10.5)
Participants consuming all five food groups		94 (31.7)
	Consuming at least one vegetable	261 (88.2)
	Consuming at least one fruit	212 (71.6)
	Consuming at least one pulse/nut/seed	140 (47.3)
	Consuming at least one animal-source food	289 (97.6)
	Consuming at least one starchy staple food	258 (87.2)

**Table 3:** Cohen’s kappa coefficients for DQQ items; data shown as mean (95% CI).

DQQ Item	Cohen’s Kappa
Q1 Pasta, ravioli or tortellini, rice, bread, focaccia, pizza, piadina, or crackers?	0.72 (0.57; 0.87)
Q2 Barley, spelt, whole grain pasta, polenta, muesli, or popcorn?	0.83 (0.69; 0.96)
Q3 Potatoes or gnocchi?	0.95 (0.89; 1.00)
Q4 Beans, chickpeas, fava beans, lentils, peas, tofu, or soymilk?	0.88 (0.77; 0.99)
Q5 Carrots, pumpkin, or red bell pepper?	0.90 (0.81; 0.99)
Q6.1 Spinach, kale, arugula, escarole, chard, watercress, or chicory?	0.86 (0.73; 0.98)
Q6.2 Basil pesto, broccoli rabe, or broccoli?	0.91 (0.79; 1.00)
Q7.1 Tomatoes, eggplant, zucchini, mushrooms, radicchio, fennel, or lettuce?	0.80 (0.68; 0.93)
Q7.2 Artichoke, asparagus, cabbage, cauliflower, beets, green pepper, or green beans?	0.72 (0.52; 0.91)
Q8 Apricot, dried apricot, loquat, canteloupe, or persimmon?	0.85 (0.64; 1.00)
Q9 Orange, mandarin, clementine, or grapefruit?	0.87 (0.77; 0.97)
Q10.1 Dried or fresh figs, dried or fresh plums, grapes, peaches, apple, pear, or banana?	0.77 (0.64; 0.9)
Q10.2 Watermelon, honeydew melon, kiwi, pomegranate, strawberries, cherries, or berries?	0.86 (0.72; 0.99)
Q11 Cakes, biscuits, merendine, cannoli, pastries, donuts, or tart?	0.83 (0.71; 0.94)
Q12 Candy, chocolates, gelato, semifreddo, tiramisu, panna cotta, custard, or chocolate pudding?	0.88 (0.78; 0.97)
Q13 Eggs?	0.83 (0.72; 0.94)
Q14 Cheese or fresh cheese?	0.86 (0.76; 0.97)
Q15 Yogurt?	0.94 (0.87; 1.00)
Q16 Cured meats such as prosciutto, mortadella, salted beef, salami, pork belly, or dried sausage?	0.92 (0.84; 1.00)
Q17 Beef, veal, lamb, sheep, goat, or meatballs?	0.95 (0.87; 1.00)
Q18 Pork, rabbit, or horse?	0.83 (0.68; 0.99)
Q19 Chicken or turkey?	0.84 (0.73; 0.95)
Q20 Fish, canned fish, anchovies, sardines, octopus, or other, seafood?	1.00 (1.00; 1.00)
Q21 Walnuts, hazelnuts, pistachios, almonds, cashews, or peanuts?	0.93 (0.86; 1.00)
Q22 Chips such as San Carlo, Fonzies, or Pringles?	0.96 (0.87; 1.00)
Q23 Instant Ramen?	1.00 (1.00; 1.00)
Q24 French fries, fried fish, fried vegetables, fried veal cutlet, fried meatballs, fried meat pastry, or chicken nuggets?	0.79 (0.65; 0.93)
Q25 Milk?	0.82 (0.71; 0.93)
Q26 Sweetened coffee, tea with sugar, hot chocolate, or chocolate drinks?	0.87 (0.77; 0.97)
Q27 Juice, fruit flavored drinks, or fruit syrup?	0.86 (0.74; 0.98)
Q28 Soft drinks such as Fanta, Coca-Cola, or Sprite, energy drinks, or sports drinks?	0.97 (0.91; 1.00)
Q29 McDonald’s, Burger King, KFC, or other place that serve burgers and french fries?	0.88 (0.66; 1.00)

**Table 4:** Global application od Diet Quality Questionnaire (DQQ) in different contexts.

Region/Country	Study Context/Population	Purpose/Key Use of DQQ
Rwanda [14]	Adults; validation vs. observed intake	Validation of DQQ vs. observed dietary intake
China [15]	School-aged children, adolescents, adults	Mental health (depression, anxiety), stress, cognitive decline, obesity, diet supplements, time-use mediation
Bangladesh [20]	Rickshaw pullers	Food insecurity + diet quality in informal workers
Pakistan [19]	Adolescents (cross-sectional study)	Diet quality + socioeconomic factors
Pakistan (ANEP) [21]	School-based nutrition intervention	Pre/post evaluation using DQQ
Ghana [16,17]	Adults; adolescents; pregnant teens	Diet quality determinants; food insecurity
Chile [22]	Pregnant women & children	Validation of sentinel foods; GDR indicators
Malaysia [23]	Working women	Food insecurity + diet quality
India [24]	Mothers and children	Social & behavior change communication + social protection
Ethiopia, Vietnam, Solomon Islands [18]	Women	Multi-country validation of DQQ
Multi-country (6 nations) [25]	National sentinel food performance	Robustness of sentinel lists
Global [3]	Adaptation & translation of DQQ	Harmonization for DHS, national surveys

## Data Availability

The data are anonymized and stored in an offline database under the surveillance of the authors and Poznan University of Medical Sciences.

## Abbreviations

The following abbreviations are used in this manuscript:

NCDs	Non-communicable diseases
FFQ	Food frequency questionnaires
DQQ	Diet Quality Questionnaire
HEI	Healthy Eating Index
MEDAS	Mediterranean Diet Adherence Screener
MedQ-Sus	Mediterranean Diet and Nutritional Sustainability Questionnaire, short Mediterranean diet questionnaire
KomPAN	Dietary Habits and Nutrition Beliefs Questionnaire
WOBASZ II	National Multicentre Health Survey
MDD-W	Minimum Dietary Diversity for Women
DSS	Dietary Diversity Score
GDRS	Global Dietary Recommendation Score
OWFR	Observed Weighed Food Record

## Appendix A

Polish Validated Language Adaptation of Diet Quality Questionnaire (DQQ)

Chcemy zadać Ci kilka pytań typu „tak” lub „nie” dotyczących jedzenia i picia, które spożywałeś wczoraj w ciągu dnia lub nocy—niezależnie od tego, czy w domu, czy gdzie indziej. Najpierw pomyśl o wczorajszym dniu, od momentu, gdy się obudziłeś, aż do nocy. Pomyśl o pierwszej rzeczy, którą zjadłeś lub wypiłeś po przebudzeniu rano. Pomyśl, gdzie byłeś, gdy jadłeś lub piłeś coś w środku dnia. Pomyśl, gdzie byłeś podczas wieczornego posiłku oraz o jedzeniu lub napojach, które mogłeś spożyć wieczorem lub późno w nocy i o wszelkich przekąskach lub napojach między posiłkami w ciągu dnia lub nocy. Interesuje mnie, czy spożywałeś produkty, które wymienię, nawet jeśli były częścią innych potraw. Proszę, przejrzyj listę produktów i napojów, a jeśli zjadłaś lub wypiłaś którykolwiek z nich, powiedz „tak”. Instrukcja: Odpowiedz TAK lub NIE na każde pytanie dotyczące produktów spożywanych wczoraj.

	**Odpowiedź**
1. Makaron (np. spaghetti, ravioli, tortellini), ryż, chleb, focaccia, pizza, piadina lub krakersy?	TAK/NIE
2. Jęczmień, orkisz, pełnoziarnisty makaron, polenta, musli lub popcorn?	TAK/NIE
3. Ziemniaki lub gnocchi?	TAK/NIE
4. Fasola, ciecierzyca, bób, soczewica, groszek, tofu lub mleko sojowe?	TAK/NIE
5. Marchew, dynia lub czerwona papryka?	TAK/NIE
6.1 Szpinak, jarmuż, rukola, cykoria, boćwina, rzeżucha lub endywia?	TAK/NIE
6.2 Pesto bazyliowe, brokuły lub brokuły rabe?	TAK/NIE
7.1 Pomidory, bakłażan, cukinia, pieczarki, radicchio, koper włoski lub sałata?	TAK/NIE
7.2 Karczoch, szparagi, kapusta, kalafior, buraki, zielona papryka lub fasolka szparagowa?	TAK/NIE
8. Morela, suszona morela, nieszpułka, melon kantalupa lub kaki?	TAK/NIE
9. Pomarańcza, mandarynka, klementynka lub grejpfrut?	TAK/NIE
10.1 Świeże lub suszone figi, śliwki, winogrona, brzoskwinie, jabłko, gruszka lub banan?	TAK/NIE
10.2 Arbuz, melon miodowy, kiwi, granat, truskawki, czereśnie lub jagody?	TAK/NIE
11. Ciasta, ciasteczka, słodkie bułki, cannoli, pączki lub tarty?	TAK/NIE
12. Cukierki, czekoladki, lody, semifreddo, tiramisu, panna cotta, budyń lub krem czekoladowy?	TAK/NIE
13. Jajka?	TAK/NIE
14. Ser lub świeży ser?	TAK/NIE
15. Jogurt?	TAK/NIE
16. Wędliny (np. prosciutto, mortadela, salami, suszona kiełbasa)?	TAK/NIE
17. Wołowina, cielęcina, jagnięcina, mięso kozie lub klopsiki?	TAK/NIE
18. Wieprzowina, królik lub konina?	TAK/NIE
19. Kurczak lub indyk?	TAK/NIE
20. Ryby, ryby w puszce, sardynki, anchois, ośmiornica lub inne owoce morza?	TAK/NIE
21. Orzechy włoskie, laskowe, pistacje, migdały, nerkowce lub orzeszki ziemne?	TAK/NIE
22. Chipsy (np. San Carlo, Fonzies, Pringles)?	TAK/NIE
23. Zupki instant (np. ramen)?	TAK/NIE
24. Frytki, smażona ryba, smażone warzywa, kotlet panierowany, smażone klopsiki, smażone pierogi mięsne lub nuggetsy?	TAK/NIE
25. Mleko?	TAK/NIE
26. Słodzona kawa, herbata z cukrem, gorąca czekolada lub napoje czekoladowe?	TAK/NIE
27. Sok, napoje owocowe lub syrop owocowy?	TAK/NIE
28. Napoje gazowane (np. Fanta, Coca-Cola, Sprite), napoje energetyczne lub izotoniczne?	TAK/NIE
29. McDonald’s, Burger King, KFC lub innym, gdzie serwują burgery i frytki	TAK/NIE
